# Supplementary material for: Splice-Junction-Based Mapping of Alternative Isoforms in the Human Proteome
Source: Cell Rep. Author manuscript; Available in PMC 2020 Jan 15. (PMC6961840; doi:10.1016/j.celrep.2019.11.026)
Supplement: 2 [file NIHMS1546469-supplement-2.zip › DF1/PXD006675/LeftAtrium/LeftAtrium_40_ABCG1_TVGWVGTAMNASSYSAEMTEPK.pdf]

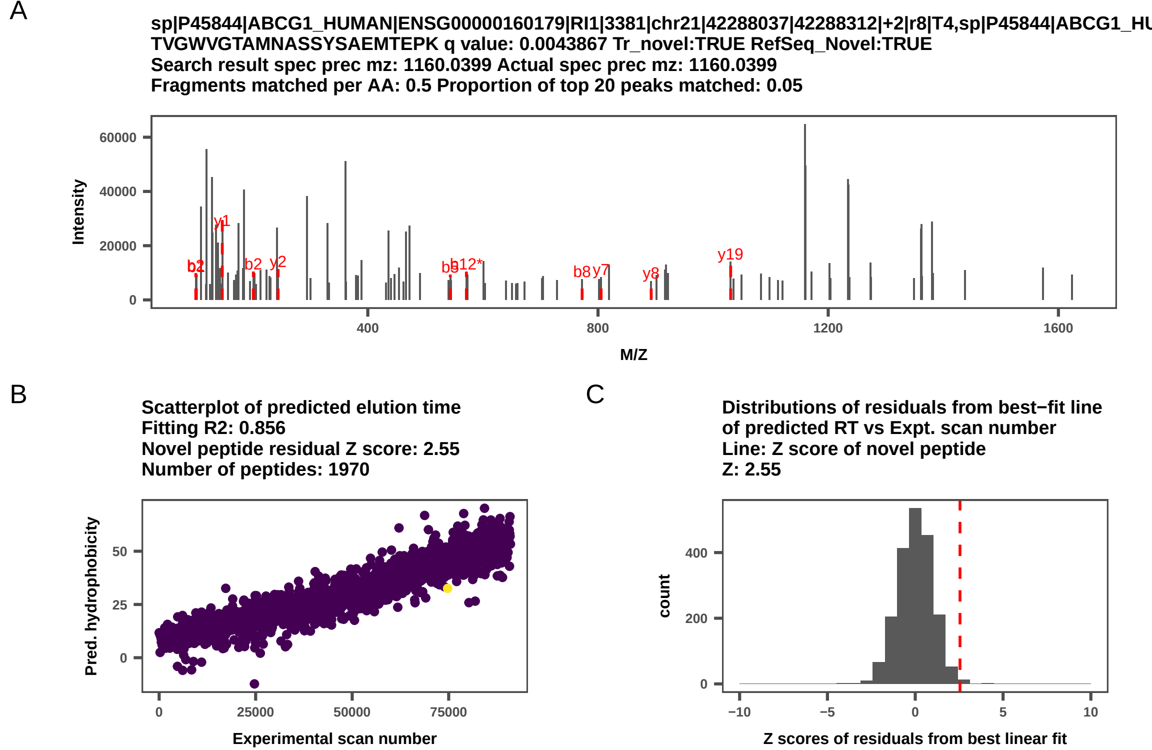
A
sp|P45844|ABCG1_HUMAN|ENSG00000160179|RI1|3381|chr21|42288037|42288312|+2|r8|T4,sp|P45844|ABCG1_HU
TVGWVGTAMNASSYSAEMTEPK q value: 0.0043867 Tr_novel:TRUE RefSeq_Novel:TRUE
Search result spec prec mz: 1160.0399 Actual spec prec mz: 1160.0399
Fragments matched per AA: 0.5 Proportion of top 20 peaks matched: 0.05
Intensity
60000
40000
20000
0
y1
b1
b2
y2
b5
b12*
b8
y7
y8
y19
400
800
1200
1600
M/Z
B
Scatterplot of predicted elution time
Fitting R2: 0.856
Novel peptide residual Z score: 2.55
Number of peptides: 1970
Pred. hydrophobicity
50
25
0
0
25000
50000
75000
Experimental scan number
C
Distributions of residuals from best−fit line
of predicted RT vs Expt. scan number
Line: Z score of novel peptide
Z: 2.55
count
400
200
0
−10
−5
0
5
10
Z scores of residuals from best linear fit
